# Evaluation of a new molecular test for the detection of SARS-CoV-2 nucleic acid in salivary samples

**DOI:** 10.5937/jomb0-43822

**Published:** 2023-10-27

**Authors:** Ilaria Talli, Andrea Padoan, Stefania Moz, Filippo Navaglia, Mario Plebani, Daniela Basso

**Affiliations:** 1 University-Hospital of Padova, Laboratory Medicine Unit, Padova, Italy; 2 Spinoff of University of Padova, QI. Lab. Med, Padova, Italy; 3 Spinoff of University of Padova, QI. Lab. Med, Padova, Italy + University of Padova, Department of Medicine-DIMED, Padova, Italy

**Keywords:** COVID-19, molecular testing, nucleic acid amplification, salivary samples, SARS-CoV-2, COVID-19, molekularno testiranje, amplifikacija nukleinske kiseline, uzorci pljuvačke, SARS-CoV-2

## Abstract

**Background:**

Molecular testing is considered the gold standard for the detection of SARS-CoV-2. This study aimed to compare the performance of the P742H SARS-CoV-2 Nucleic Acid Multiplex Detection Kit in salivary samples, with respect to the 732HF Novel Coronavirus (2019-nCoV) Nucleic Acid Detection Kit and the TaqPath COVID-19 CEIVD RT-PCR Kit, used at University-Hospital of Padova, Italy.

**Methods:**

One hundred twenty-four salivary samples selfcollected by healthcare workers (HCW) during the screening program at University-Hospital of Padova, Italy, from Oct to Nov 2022, were included in the study. RNA extraction was performed by Viral DNA and RNA Extraction Kit (Technogenetics, Lodi, Italy) and amplification by P742H and 732HF (Technogenetics, Lodi, Italy). RNA was extracted using MagNa Pure 96 DNA and Viral NA Small Volume Kit (Roche, Switzerland) for TaqPath analysis (Thermo Fisher Scientific, USA).

## Abstract (sr)

**Uvod:**

Molekularno testiranje se smatra zlatnim standardom za otkrivanje SARS-CoV-2. Ova studija imala je za cilj da uporedi performanse kompleta za detekciju multipleksa nukleinske kiseline P742H SARS-CoV-2 u uzorcima pljuvačke, u odnosu na komplet za detekciju nukleinske kiseline 732HF novog koronavirusa (2019-nCoV) i TakPath COVID-19 CE-IVD RT -PCR komplet, koji se koristi u Univerzitetskoj bolnici u Padovi, Italija.

**Metode:**

U studiju je uključeno 124 uzorka pljuvačke koje su sami sakupili zdravstveni radnici (ZR) tokom programa skrininga u Univerzitetskoj bolnici u Padovi, Italija, od oktobra do novembra 2022. Ekstrakcija RNK je izvedena pomoću kompleta za ekstrakciju virusne DNK i RNK (Technogenetics, Lodi, Italija), a amplifikacija pomoću P742H i 732HF (Technogenetics, Lodi, Italija). RNK je ekstrahovana koriščenjem MagNa Pure 96 DNK i Viral NA Small Volume Kit (Roche, vajcarska) za TakPath analizu (Thermo Fisher Scientific, SAD).

**Rezultati:**

94 uzorka bila su pozitivna na P742H, dok je 30 bilo negativno; za 732HF, 96 uzoraka je bilo pozitivno, dok je 28 bilo negativno, sa ukupnim slaganjem od 97,5% (Koenov = 0,930, p < 0,001). TakPath je dao 95 pozitivnih uzoraka i 29 negativnih rezultata, sa ukupnim slaganjem od 100% (Koenov = 1,0, p < 0,001) u odnosu na P742H, i 97,5% (Koenov = 0,931, p < 0,0032) u odnosu na HF od 97,5%. Upoređujuči prag ciklusa (Ct) između P742H i 732HF, nisu pronađene statistički značajne razlike (p = n.s.).

**Zaključak:**

Metoda P742H pokazala je bolje performanse od 732HF za uzorke pljuvačke, pri čemu su oba pokazala isto vreme amplifikacije. Pored toga, rezultati P742H bili su uporedivi sa onima dobijenim metodom visoke propusnosti TakPath.

## Introduction

For a long time, COVID-19 has been a worldwide health problem, for political, social, and clinical reasons, and therefore it has been treated with emergency dispositions. Thanks to the development of vaccines and the subsequent vaccine campaigns, the pandemic emergency had an arrest, reaching a higher population immunity globally. The World Health Organization (WHO) Emergency Committee agreed that the COVID-19 pandemic might be reaching an inflexion point, while still being a clinical issue that continues to have an impact on morbidity and mortality [1]. Nevertheless, the diagnosis of infected subjects remains important especially in fragile patients, to prevent the clinical complications and pulmonary involvement, while screening strategies may be of relevance in specific contexts, such as in hospital settings and healthcare workers (HCW) monitoring, to reduce the spread of the infection [2] [3] [4].

The gold standard method for diagnosis of SARS-CoV-2 infection is based on the nucleic acid amplification tests (NAAT). Real-time PCR enables the detection and quantification of viral genes [5]. The analysis of the presence of RNA viral sequences started with the extraction of nucleic acid from nasopharyngeal swabs (NPS) or saliva samples, followed by reverse transcription into cDNA and then by PCR amplification. The outcome is given in terms of positivity or negativity of infection based on the threshold cycle that results from the analysis of each gene analyzed in the panel, which could be automatically detected by advanced software [6] [7]. Other laboratory methods, such as immunoassays, are used to rapidly test SARS-CoV-2 presence in patients' samples [8]. Recently, there has been proving evidence that NAAT integration with immunoassays detecting SARS-CoV-2 viral antigens could represent a promising cost-effective strategy for confining COVID-19 spread. However, in some circumstances requiring high sensitivity and specificity (e.g. patients receiving organ transplant, medical surgery, elderly fragile people), the utilization of NAAT can be favored with respect to antigen testing. Indeed, in a recent meta-analysis, it was demonstrated that rapid antigen tests using nasal or NPS showed a steady decline in sensitivity as the measures of sample viral load decline; the average sensitivity ranged from 34.3% to 91.3% in symptomatic participants, and from 28.6% to 77.8% for asymptomatic subjects [9]. These results underlined that rapid antigen tests can be useful in detecting positivity of individuals with high viral load, while sensitivity remains too low for other settings [9].

In addition to different laboratory procedures, a variety of biological matrices can be used. Patients' sampling is done through NPS collection, especially for individuals with high tract respiratory infections. However, NPS have some drawbacks that must be considered. First of all, they cannot be performed individually by patients themselves, but they require expert HCW [10]; secondly, they require the use of adequate personal protecting equipment to protect HCW, and they also may facilitate viral spread between individuals who undergo testing. Saliva is a valid alternative for several reasons, having the advantage of being self-collected by patients and enabling an easy sample handling [11]. Moreover, saliva collection is non-invasive and can enhance patients' compliance and simplicity of collection, especially in screening settings [8] [12], in addition to reducing the total costs by 25-30% with respect to NPS [13]. Notably, some recent studies highlighted saliva results were concordant with NPS results both in qualitative and quantitative terms [8] [12] [13] [14]. Differently, for antigen detection rapid diagnostic test (Ag-RDT), saliva was described as less sensitive with respect to NPS [15]. Furthermore, SARS-CoV-2 antigen levels in saliva decrease more rapidly than in NPS when analyzed with respect to the decline in viral load [8].

The aim of this study was to compare the performances of a new fast molecular method to detect the presence of SARS-CoV-2 in salivary samples of patients, the Technogenetics SARS-CoV-2 Nucleic Acid Multiplex Detection Kit (P742H), useful also as a confirmatory test for screening programs, with respect to two methods for NAAT, one from the same manufacturer and the other one in use at University-Hospital of Padova, Italy (AOPD).

## Materials and methods

### Samples included in the analysis

For the aim of the study, 124 (43 males and 81 females) leftover salivary samples were randomly selected from the HCW ongoing screening program at AOPD, between October 24^th^ 2022 and November 21^st^ 2022. Samples were self-collected using Salivette® (Sarstedt, Germany), centrifuged for 5 minutes at 4000g, then tested for SARS-CoV-2 for the screening program (as specified below) and, after that, immediately stored at-80°C until use.

### Extraction and amplification procedures

The 124 samples were analyzed in two analytic sessions, with half of the samples each, on the 17^th^ November and 24^th^ November 2022 respectively. Extraction and amplification were executed on the same day. The obtained results were subsequently compared with the respective results obtained from the ongoing screening program.

After thawing samples at room temperature for30 minutes, salivary samples underwent RNA extraction through the use of two extraction kits. The firstextraction was performed using Viral DNA and RNAExtraction Kit (REF T014H version 1), provided byTechnogenetics (Lodi, Italy), following manufacturer’srecommendations with a dedicated Nucleic AcidExtractor machine (provided by Technogenetics). Thesecond RNA extraction was performed with MagNAPure 96 DNA and Viral NA Small Volume Kit (REF06543588001 version 09) (Roche, Switzerland) inthe nucleic acid extractor Magna Pure 96 (Roche)and then analyzed using the method used at AOPDfor HCW screening program.

RNAs extracted with the first method were amplified using the two different kits, the SARS-CoV-2 Nucleic Acid Multiplex Detection Kit (REF P742H version 3.0) (P742H) and the Novel Coronavirus (2019-nCoV) Nucleic Acid Detection Kit (REF P732HF version 1.0) (732HF), both provided by Technogenetics using a real-time PCR in Gentier 96 thermocycler (Technogenetics). Both amplification reactions are real-time PCRs that exploit TaqMan probes for the detection of the different genes included in the assay. The SARS-CoV-2 Nucleic Acid Multiplex Detection Kit (P742H) enables the recognition of three candidate genes and one internal control (IC) gene, while the Novel Coronavirus (2019-nCoV) Nucleic Acid Detection Kit (732HF) is based on the detection of two genes and one IC gene. The IC gene was used as a control of extraction and amplification reaction performances: it should give positive results for the samples to be included in the analysis. More specifically, the genes included in the P742H were *RdRp gene, N gene* and *E gene*, while the genes included in the 732HF were *ORF1ab gene* and *N gene*. For both kits, the amplification mix included a Reaction Solution, an Enzyme Mix and a Primer and Probe Mix. The amplification reaction was carried with 20 µL of amplification mix and 5 µL of RNA sample; each kit had a reaction profile of 45 amplification cycles. Moreover, each kit included one positive control and one negative control to confirm both the assays gave correct results. After the amplification reaction, results were obtained from the analysis software. Each sample was considered valid if the internal control gene turned positive; moreover, each sample was considered either positive or negative for the analysis if the genes were amplified before or after cycle 43 respectively. For both P742H and 732HF, samples with negative IC and negative amplified genes were considered invalid.

Moreover, results were compared to those obtained through the method used at AOPD for screening program. The amplification reaction was carried out using TaqPath^TM^ COVID-19 RT-PCR Kit (Applied Biosystems, Thermo Fisher Scientific, Massachusetts, USA) in QuantStudio5 thermocycler (Applied Biosystems, Thermo Fisher Scientific). The amplification reaction was a real-time PCR with TaqMan probes for the detection of the different genes included in the assay, which were *ORF1ab gene, N gene* and *S gene*. The amplification mix included the TaqPath 1-step Multiplex Master Mix (4X) and the COVID-19 Multiplex (20X), an Enzyme Mix and a Primer and Probe Mix. The amplification reaction was carried with 6 µL of amplification mix and 14 µL of RNA sample; the kit had a reaction profile of 40 amplification cycles. Moreover, two positive controls and one negative control were included to confirm the results of the assay. After the amplification reaction, results were obtained from the analysis software. Each sample was considered positive for the analysis if the genes were amplified before cycle 33. In order to test the appropriateness of salivary samples, the amplification of *RNaseP gene* was performed at the same time in the method routinely used at AOPD. Preliminary sample processing was the same, but the amplification reaction was specific. More in detail, 14 µL of extracted RNA were amplified with 8 µL of reaction mix, which contained home-designed Primers and Probes RNaseP mix (20X), AgPath Buffer (2X) and AgPath (AgPath-ID^TM^ One-Step RT-PCR Kit) (Applied Biosystems, Thermo Fisher Scientific) in QuantStudio5 thermocycler.

### Statistical analyses

All statistical analyses were performed by Stata v 16.2 (StataCorp, Lakeway drive, TX, USA). Median was used as descriptive statistics of Ct quantitative data. Cohen's kappa was used to measure interrater agreement. The module »diagt« was used to calculate sensitivity and specificity, and their 95% confidence intervals (95%CI).

### Ethical statement

The study was conducted in accordance with the Declaration of Helsinki, and the Institutional Review Board of the University of Padova (protocol no.27444).

## Results

Analyzing the results obtained through the SARS-CoV-2 Nucleic Acid Multiplex Detection Kit (P742H), 94/124 (75.8%) samples were positive for all genes RdRp, N and E, while 30/124 (24.2%) were negative; as for the IC, 108/124 (87.1%) samples were positive, while 16/124 (12.9%) were negative (Table 1). One sample (0.8%) resulted as invalid. For the Novel Coronavirus (2019-nCoV) Nucleic Acid Detection Kit (732HF), 96/124 (77.4%) samples were positive for all genes ORF1ab and N, while 28/124 (22.6%) gave a negative result; as for the IC, 120/124 (96.8%) samples were positive, while 4/124 (3.2%) were negative (Table 1). Four samples (3.2%) resulted as invalid. Analyzing the results obtained through the routine method used at AOPD (TaqPath), 95/124 (76.6%) samples were positive for genes ORF1ab and N, while 29/124 (23.4%) gave a negative result. The frequency of positive samples for the S gene is lower, since from April 2021 the TaqPath test resulted in S gene dropout in samples with a variant carrying the 69-70del mutation, as declared by Thermo Fisher Scientific (Thermofisher communication. The S gene advantage TaqPath COVID-19 tests may help early identification of B.1.17) (Table 1).

**Table 1:** Number and percentages of positive (POS) and negative (NEG) samples for the genes analyzed with P742H (SARS-CoV-2 Nucleic Acid Multiplex Detection Kit, Technogenetics), 732HF (Novel Coronavirus (2019-nCoV) Nucleic Acid Detection Kit, Technogenetics) and TaqPath amplification method.

	P742H				732HF			TaqPath		
	RdRp gene	N gene	E gene	IC	ORF1ab<br>gene	N gene	IC	ORF1ab<br>gene	N gene	S gene
POS<br>(n, (%))	94, <br>(75.8%)	94,<br>(75.8%)	94,<br>(75.8%)	108,<br>(87.1%)	96,<br>(77.4%)	96,<br>(77.4%)	120,<br>(96.8%)	95,<br>(76.6%)	95,<br>(76.6%)	7,<br>(5.6%)
NEG<br>(n, (%))	30, <br>(24.2%)	30,<br>(24.2%)	30,<br>(24.2%)	16,<br>(12.9%)	28,<br>(22.6%)	28,<br>(22.6%)	4,<br>(3.2%)	29,<br>(23.4%)	29,<br>(23.4%)	117,<br>(94.4%)

The agreement between the different amplification methods, highlighting the number of positive (P), negative (N) and invalid (I) samples obtained for each assay were reported in Table 2.

**Table 2:** Comparison of the results obtained through the three amplification methods, highlighting the positive (P), negative (N) and invalid (I) samples for each method.

		P742H					TaqPath				TaqPath	
		P	N	I			P	N			P	N
732HF	P	89	2	1	P742H	P	94	0	732HF	P	90	2
	N	1	27	0		N	0	29		N	1	27
	I	4	0	0		I	1	0		I	4	0

Between methods agreements were calculated by excluding invalid results. From the comparison between P742H and 732HF, the agreement was 97.5%, with a Cohen's kappa of 0.930 (SE= 0.092, z= 10.2, p < 0.001). From the comparison between P742H and TaqPath, the agreement was 100%, with a Cohen's kappa of 1.0 (SE= 0.090, z= 11.1, p < 0.001). From the comparison between 732HF and TaqPath, the agreement was 97.5%, with a Cohen's kappa of 0.931 (SE= 0.0913, z= 10.2, p < 0.001).

Using TaqPath as the reference method, P742H and 732HF sensitivity and specificity values were estimated excluding the invalid samples from the analyses. For P742H, the sensitivity and specificity were 100% (95%CI: 96.2%-100%) and 100% (95%CI: 88.1%-100%), respectively, with a positive and negative likelihood ratio of 59.7 (95%CI: 3.82-932.4) and 0.01 (95%CI: 0.01-0.08). For 732HF, the sensitivity and specificity were 98.9% (95%CI: 94.0%-100%) and 93.1 (95%CI: 77.2%-99.2%), respectively, with a positive and negative likelihood ratio of 14.3 (95%CI: 3.8-54.6) and 0.01 (95%CI: 0.01-0.08).

**Figure 1:**
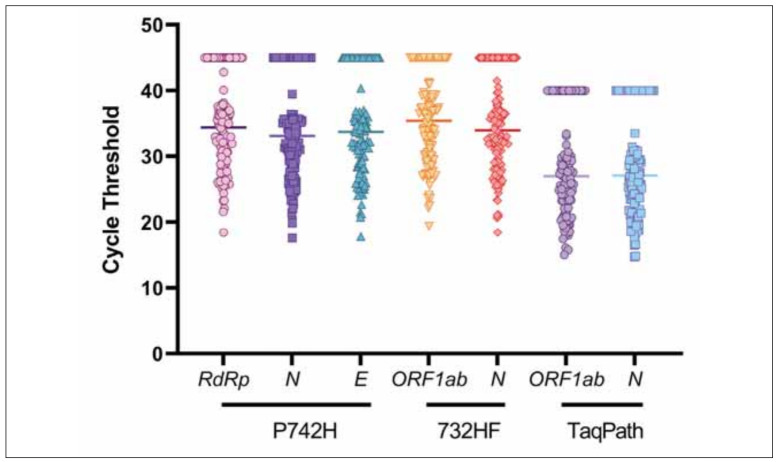
Threshold cycles (Ct) for all samples for the genes analyzed with P742H, 732HF and TaqPath assay, highlighting the median value for each gene. Maximum Ct values were 45 for P742H and 732HF and 40 for TaqPath.

Figure 1 shows the threshold cycles (Ct) values of P742H, 732HF and TaqPath assays for all the evaluated genes. The Kruskall-Wallis test, adjusted by Dunn's method for multiple testing, underlined that differences of Ct values exist only between the two TaqPath genes and all the others genes of P742H and 732HF (p < 0.001 for all). There were not statistically significant differences across median Ct values of P742H and 732HF genes.

## Discussion

Despite the national and international efforts for controlling and limiting the spread of SARS-CoV-2, COVID-19 still permanently remains an established infection in humans for a long time. The urgent need of rapid diagnosis of infected subjects is pivotal in order to limit viral spread and prevent deaths of fragile subjects. NAATs are the gold standard methods for SARS-CoV-2 molecular detection, because of their elevated analytical sensitivity and specificity [16]. Lippi *et al.* pointed out some challenges in providing routine molecular SARS-CoV-2 tests for screening contexts, especially using NPS. Difficulties can be attributed to recruiting staff for collecting NPS, and to obtaining the supplies needed for a large number of tests [17] [18]. Therefore, alternative sampling procedures have been tested. Self-collecting saliva was demonstrated to have comparable sensitivity and specificity to NPS [19] [20], in addition to being a cost-effective and a simple way of specimen collection, thus enabling accurate large-scale SARS-CoV-2 surveillance testing [20].

Furthermore, in subjects with higher viral load, in patients with critical conditions or waiting hospitalization at the emergency department, providing rapid results could be important. Molecular testing typically might require up to 4-6 hours to be completed [21]. In addition, it possibly leaves laboratories with a huge backlog of samples to be processed when an elevated number of tests are required simultaneously [17]. The implementation of novel rapid molecular tests may help to overcome the challenges described above, especially when patients' conditions require it. In addition, it has been demonstrated that strategies based on antigen (Ag) test, performed with laboratory equipment, coupled with real-time PCR testing in case of a first negative result offer better performances than Ag test alone, with a decreased cost with respect to real-time PCR alone [22].

In the present study, the SARS-CoV-2 Nucleic Acid Multiplex Detection Kit (P742H) has been evaluated and tested with respect to the Novel Corona virus (2019-nCoV) Nucleic Acid Detection Kit (732HF). After an RNA extraction (around 20 minutes), both assays, supplied by Technogenetics, require 60 minutes for giving qualitative (pos/neg) and quantitative (genes Ct) results for 64 samples. The two assays were further compared with a the TaqPath assay, routinely used at AOPD and, thus, considered as a reference method. Both assays were tested using 124 samples (95/124 positive, 76.6% for TaqPath assay); for P742H and 732HF one and four samples resulted as invalid, respectively, and the agreement was 97.5% (3 samples were discordant), with a Cohen's kappa of 0.930. Two of these samples were negative and one positive at TaqPath (which was exactly comparable to P742H). The efficiency in probe and primer design may affect the system performance, in addition to the amount of sample collected and the input volume of the specimen [23]. Moreover, misinterpretation of samples may happen also because the amount of virus is below the detection limit of the method, leading to failure to detect a positive result [23]. However, as pointed out by Fomenko *et al.* [24], for Ct above 35, the probability of obtaining a positive viral culture in infected subjects is very limited, virtually equal to zero for most of the molecular tests.

At last, fully automated systems ensure a more precise and standardized handling and testing of large numbers of samples, while manual work intrinsically carries HCW-specific limits [23]. Indeed, from the quantitative Ct analysis of studied genes, no significant differences were found.

This study also included some limitations, such as the number of evaluated specimens, which was not elevated. However, the samples were evaluated with three different assays, confirming the solidity of the results. A further limitation is the lack of NPS comparison for individuals included in the study. However, salivary SARS-CoV-2 testing by TaqPath method was previously validated in a cohort of 6284 subjects, including 206 individuals with COVID-19, with positivity confirmed by NPS [13].

## Conclusion

The development of novel, fast molecular kits for the diagnosis of SARS-CoV-2 infection is needed for the rapid and accurate identification of infected individuals [23] [25]. The SARS-CoV-2 Nucleic Acid Multiplex Detection Kit (P742H) resulted accurate and fast to be applied not only in high-risk individuals, but also in situations requiring a rapid and am accurate diagnosis of COVID-19.

## Dodatak

### Acknowledgment

The Authors thank Technogenetics for kindly supplying reagents without in any way influencing the study design and data analysis.

### Conflict of interest statement

All the authors declare that they have no conflict of interest in this work.

## References

[1] 1. *** (2005). Statement on the fourteenth meeting of the International Health Regulations (2005) Emergency Committee regarding the coronavirus disease (COVID-19) pandemic. https://www.who.int/news/item/30-01-2023-statement-on-the-fourteenth-meeting-of-the-international-health-regulations-(2005)-emergency-committee-regarding-the-coronavirus-disease-(covid-19)-pandemic.

[2] Thomas Craig K J, Rizvi R, Willis V C, Kassler W J, Jackson G P (2021). Effectiveness of contact tracing for viral disease mitigation and suppression: Evidence-based review. JMIR Public Health Surveill.

[3] Citak N, Pekcolaklar A (2021). COVID-19 screening program should be performed in healthcare workers. Turkish Thorac J.

[4] Udani R, Schilter K F, Hillmer R E, Petersen R A, Srinivasan S, Marchant J S, et al (2022). Implementation of an active screening program for SARS-CoV2: Experience at an academic medical center. WMJ.

[5] Wilhelm A, Pallas C, Marschalek R, Widera M (2022). Detection and quantification of SARS-CoV-2 By real-time RT-PCR assay. Methods Mol Biol.

[6] 6. *** (2020). WHO Coronavirus disease (COVID-19) technical guidance: Laboratory testing for 2019-nCoV in humans. https://www.who.int/emergencies/diseases/novelcoronavirus-2019/technical-guidance/laboratory-guidance.

[7] 7. World Health Organization (2 March 2020). Laboratory testing for coronavirus disease 2019 (COVID-19) in suspected human cases: Interim guidance. https://apps.who.int/iris/handle/10665/331329.

[8] Basso D, Aita A, Padoan A, Cosma C, Navaglia F, Moz S, et al (2021). Salivary SARS-CoV-2 antigen rapid detection: A prospective cohort study. Clin Chim Acta.

[9] Dinnes J, Sharma P, Berhane S, van Wyk S S, Nyaaba N, Domen J, et al (2022). Rapid, point-of-care antigen tests for diagnosis of SARS-CoV-2 infection. Cochrane Database Syst Rev.

[10] Lippi G, Henry B M, Plebani M (2022). An overview of the most important preanalytical factors influencing the clinical performance of SARS-CoV-2 antigen rapid diagnostic tests (Ag-RDTs). Clin Chem Lab Med.

[11] Bastos M L, Perlman-Arrow S, Menzies D, Campbell J R (2021). The sensitivity and costs of testing for SARS-CoV-2 infection with saliva versus nasopharyngeal swabs: A systematic review and meta-analysis. Ann Intern Med.

[12] Aita A, Basso D, Cattelan A M, Fioretto P, Navaglia F, Barbaro F, et al (2020). SARS-CoV-2 identification and IgA antibodies in saliva: One sample two tests approach for diagnosis. Clin Chim Acta.

[13] Basso D, Aita A, Navaglia F, Mason P, Moz S, Pinato A, et al (2022). The University of Padova salivary-based SARS-CoV-2 surveillance program minimized viral transmission during the second and third pandemic wave. BMC Med.

[14] Butler-Laporte G, Lawandi A, Schiller I, Yao M, Dendukuri N, McDonald E G, Lee T C (2021). Comparison of saliva and nasopharyngeal swab nucleic acid amplification testing for detection of SARS-CoV-2: A systematic review and meta-analysis. JAMA Intern Med.

[15] Schuit E, Venekamp R P, Veldhuijzen I K, van den Bijllaardt W, Pas S D, Stohr J J J M, et al (2022). Head-to-head comparison of the accuracy of saliva and nasal rapid antigen SARS-CoV-2 self-testing: Cross-sectional study. BMC Med.

[16] Rhoads D D, Pinsky B A (2021). The truth about SARS-CoV-2 cycle threshold values is rarely pure and never simple. Clin Chem.

[17] Lippi G, Henry B M (2022). Possible drawbacks of relying only on molecular testing for diagnosing SARS-CoV-2 infections. Public Health.

[18] Bohn M K, Lippi G, Horvath A, Sethi S, Koch D, Ferrari M, et al (2020). Molecular, serological, and biochemical diagnosis and monitoring of COVID-19: IFCC taskforce evaluation of the latest evidence. Clin Chem Lab Med.

[19] Yokota I, Shane P Y, Okada K, Unoki Y, Yang Y, Inao T, et al (2021). Mass screening of asymptomatic persons for severe acute respiratory syndrome coronavirus 2 using saliva. Clin Infect Dis.

[20] Rao M, Rashid F A, Sabri F S A H, Jamil N N, Zain R, Hashim R, et al (2021). Comparing nasopharyngeal swab and early morning saliva for the identification of severe acute respiratory syndrome Coronavirus 2 (SARS-CoV-2). Clin Infect Dis.

[21] Amrita J, Pal Singh A (2022). Role of arterial blood gas (ABG) as a valuable assessment tool of disease severity in SARS-CoV-2 patients. J Med Biochem.

[22] Pighi L, Henry B M, Mattiuzzi C, De Nitto S, Salvagno G L, Lippi G (2023). Cost-effectiveness analysis of different COVID-19 screening strategies based on rapid or laboratory-based SARS-CoV-2 antigen testing. Clin Chem Lab Med.

[23] You H L, Lin M C, Lee C H (2021). Comparison of the Roche cobas 6800 SARS-CoV-2 test and the Taiwan CDC protocol for the molecular diagnosis of COVID-19. Biomed J.

[24] Fomenko A, Weibel S, Moezi H, Menger K, Schmucker C, Metzendorf M I, et al (2022). Assessing severe acute respiratory syndrome coronavirus 2 infectivity by reverse-transcription polymerase chain reaction: A systematic review and meta-analysis. Rev Med Virol.

[25] Banko A, Petrovic G, Miljanovic D, Loncar A, Vukcevic M, Despot D, et al (2021). Comparison and sensitivity evaluation of three different commercial real-time quantitative PCR kits for SARS-CoV-2 detection. Viruses.

